# Effect of Aging and Cooling Path on the Super β-Transus Heat-Treated Ti-6Al-4V Alloy Produced via Electron Beam Melting (EBM)

**DOI:** 10.3390/ma15124067

**Published:** 2022-06-08

**Authors:** Alessandro Carrozza, Giulio Marchese, Abdollah Saboori, Emilio Bassini, Alberta Aversa, Federica Bondioli, Daniele Ugues, Sara Biamino, Paolo Fino

**Affiliations:** 1 Department of Applied Science and Technology, Politecnico di Torino, C.so Duca degli Abruzzi 24, 10129 Turin, Italy; giulio.marchese@polito.it (G.M.); emilio.bassini@polito.it (E.B.); alberta.aversa@polito.it (A.A.); federica.bondioli@polito.it (F.B.); daniele.ugues@polito.it (D.U.); sara.biamino@polito.it (S.B.); paolo.fino@polito.it (P.F.); 2 Consorzio Interuniversitario Nazionale per la Scienza e Tecnologia dei Materiali (INSTM), Via Giuseppe Giusti 9, 50121 Florence, Italy; 3 Department of Management and Production Engineering, Politecnico Di Torino, C.so Duca degli Abruzzi 24, 10129 Turin, Italy; abdollah.saboori@polito.it

**Keywords:** additive manufacturing, electron beam melting, titanium, heat treatments, microstructure, mechanical properties

## Abstract

This work focuses on the effect of different heat treatments on the Ti-6Al-4V alloy processed by means of electron beam melting (EBM). Super β-transus annealing was conducted at 1050 °C for 1 h on Ti-6Al-4V samples, considering two different cooling paths (furnace cooling and water quenching). This heat treatment induces microstructural recrystallization, thus reducing the anisotropy generated by the EBM process (columnar prior-β grains). Subsequently, the annealed furnace-cooled and water-quenched samples were aged at 540 °C for 4 h. The results showed the influence of the aging treatment on the microstructure and the mechanical properties of the annealed EBM-produced Ti-6Al-4V. A comparison with the traditional processed heat-treated material was also conducted. In the furnace-cooled specimens consisting of lamellar α+β, the aging treatment improved ductility and strength by inducing microstructural thickening of the α laths and reducing the β fraction. The effect of the aging treatment was also more marked in the water-quenched samples, characterized by high tensile strengths but limited ductility due to the presence of martensite. In fact, the aging treatment was effective in the recovery of the ductility loss, maintaining high tensile strength properties due to the variation in the relative number of α/α’ interfaces resulting from α’ decomposition. This study, therefore, offers an in-depth investigation of the potential beneficial effects of the aging treatment on the microstructure and mechanical properties of the EBM-processed super β-transus heat-treated Ti-6Al-4V alloy under different cooling conditions.

## 1. Introduction

Titanium alloys are essential materials in multiple industrial fields such as the aerospace, automotive, medical, and chemical sectors. These alloys are mostly considered for structural applications due to some key properties, such as biocompatibility, excellent corrosion resistance, and high specific strength [1,2,3,4]. For the sake of weight reduction and lower fuel consumption, specific strength is the main reason titanium alloys are applied in the aerospace industry, which is by far their most important consumer [5,6]. Ti-6Al-4V is the most used α+β titanium alloy, accounting for the largest share of the titanium market [7,8,9]. However, this alloy is expensive due to the high cost of the minerals, refinement technologies, and manufacturing operations (e.g., casting, forging, machining) [10,11]. Therefore, any manufacturing technique that can provide finished/semi-finished titanium products with little to no machining required appears to be a very attractive and potentially cost-effective. Thus, additive manufacturing (AM) technologies are appealing for the Ti-6Al-4V alloy, allowing the possibility of reducing the consumption of the starting material (typically powder) that can be reused for the fabrication of new components. Moreover, AM processes enable the production of complex-shaped components [12].

Among the AM technologies, electron beam melting (EBM) has been widely employed to produce components made of Ti-6Al-4V alloy, attracting the attention of industrial fields like the aerospace sector [13]. EBM processing implies the micro-melting of small areas of partially sintered feedstock powder, which provides unique microstructures. If the process is carefully planned, i.e., with a proper choice of process parameters, these can result in excellent combinations of mechanical properties for the Ti-6Al-4V alloy, even in the as-built state [13,14]. This is mainly related to the high thermal gradients and cooling rates developed, which result in the formation of fine columnar grains [15]. Moreover, the long duration of the process, in a chamber kept at approximately 700 °C [13,16], favors diffusive phenomena, thus decomposing the embrittling α’ martensite into optimal α+β lamellar microstructures [17]. Furthermore, the EBM technology can be employed to produce gradient and lattice structures with excellent mechanical properties [18,19].

Post-processing heat treatments are a useful tool to specifically tailor the microstructure and mechanical properties according to the application needs. In general, the most important factor to consider when heat treating the Ti-6Al-4V alloy is its β-transus temperature (T_β_), over which the microstructure is 100% β phase (≈995–1000 °C in Ti-6Al-4V). The most common categories of heat treatments are stress-relieving, annealing, and annealing followed by aging treatment. Stress-relieving is typically avoided in EBM-produced Ti-6Al-4V since the material is kept at approximately 700 °C inside the machine during the building process [17,20]. Annealing can be conducted at temperatures slightly above T_β_ (approximately 1000 °C), allowing the microstructure to recrystallize, thus removing the columnar grain morphology, which causes mechanical properties anisotropy [21,22,23]. Conversely, annealing treatments at temperatures below the T_β_ preserve the fine grains obtained during the EBM process, although the anisotropy is retained. After the annealing step, aging can be performed. It is usually carried out at lower temperatures and for longer durations in order to promote diffusive phenomena, thus favoring the decomposition of martensite and/or supersaturated retained β phase [24].

Numerous studies in the literature focus on the post-processing heat treatments of the Ti-6Al-4V alloy produced via EBM, considering different combinations of time, temperature, and cooling medium. For instance, Raghavan et al. [25] studied different annealing heat treatments on the Ti-6Al-4V alloy, from 1 to 3 h with temperatures ranging from 930 (sub β-transus) to 1050 °C (super β-transus). Moreover, the authors considered an additional aging step for the samples annealed at 1050 °C, followed by an air cooling step. This combination of heat treatments aimed to coarsen and remove the residual martensite formed upon cooling after the annealing. The resulting mechanical properties for the last heat treatment were comparable overall, if not inferior to the as-built samples. De Formanoir et al. [26] investigated post hot isostatic pressing annealing at 950 and 1040 °C, finding substantial microstructural changes in the samples heat-treated above T_β_. In particular, they reported that the grain morphology shifted from columnar to equiaxed. However, a subsequent aging step was not considered. By contrast, Soundarapandiyan et al. [27] and Evtushenko et al. [28] evaluated the effect of an aging step on sub β-transus annealed samples rapidly cooled, which resulted in a reduction in hardness and tensile strength due to microstructural coarsening. Galarraga et al. [16] instead studied the effect of several heat treatments on Ti-6Al-4V samples produced using a mix of 50% virgin and 50% used powders. The authors considered all the main possible cooling paths: furnace, air, and water cooling. Overall, the first two led to mainly α+β microstructures, characterized by a good balance of mechanical properties. Instead, the water-quenched samples provided a mainly martensitic microstructure characterized by outstanding strength but limited ductility. Moreover, the authors also investigated several aging heat treatments exclusively applied to samples that previously underwent sub β-transus annealing. One of the most important conclusions drawn by the authors stated that annealing followed by aging is an optimal methodology in order to tailor the mechanical properties via proper time, temperature, and cooling rate adjustments.

Although several works focused on super β-transus annealings [29,30,31,32], to the best of the authors’ knowledge, little attention has been paid to the effect of aging treatments on the EBM-produced Ti-6Al-4V alloy. Thus, the present paper investigates the effect of an aging treatment (A) on the microstructure and mechanical performance of a super β-transus annealed Ti-6Al-4V alloy produced by EBM. The annealed treatment was followed by either slow furnace cooling (FC) or water quenching (WQ) in order to study also the influence of different cooling paths.

## 2. Materials and Methods

**Figure 1:**
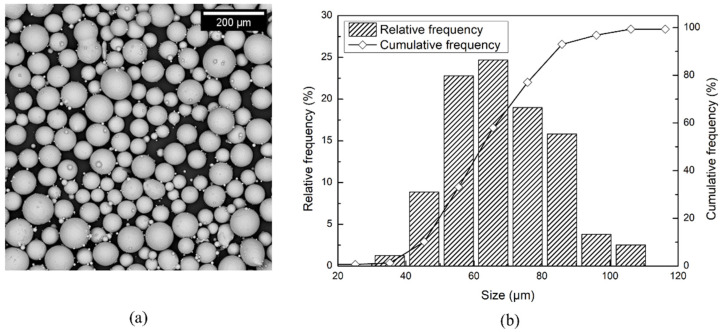
Representative image of the Ti-6Al-4V ELI powder (**a**) and related particle size distribution (**b**).

In this work, 15 × 15 × 15 mm^3^ cubes and 15 vertical cylindrical bars of 15 mm diameter and 140 mm length were built via EBM using an Arcam A2X machine (Arcam AB, Mölndal, Sweden) and a Ti-6Al-4V ELI (Extra Low Interstitials) powder as feedstock material. The specimens were produced adopting the standard process parameters given by the machine supplier. The starting powder was characterized by spherical particles (Figure 1a), and its particle size distribution (Figure 1b), evaluated by image analysis, provided a D(10), D(50), and D(90) value of 45, 62, and 84 µm, respectively.

The printed samples were heat-treated, thus providing a total of five conditions to be analyzed, as summarized in Table 1. The annealing heat treatments of the WQ and WQ+A samples were conducted in a Nabertherm RHTC 80-710/15 tube furnace (Nabertherm GmbH, Lilienthal, Germany), using a protective argon flow of 1.5 L/min in order to prevent oxidation/interstitials pick-up in the specimens. At the end of the isothermal heat treatment cycle, the samples were quickly removed from the furnace and immediately quenched in water. Temperature monitoring was performed using a type K thermocouple, characterized by an accuracy of around ±2 °C. Instead, the FC, FC+A, and all the aging steps were all conducted in a Pro.Ba VF800/S high-vacuum furnace (Pro.Ba., Cambiano, Italy), in which the samples were left to cool at the end of the heat treatment, achieving a cooling rate of approximately 1.5–2 °C/min. Temperature monitoring was achieved using a set of S thermocouples (accuracy of ±1 °C) located close to the samples. The super β-transus heat treatments were conducted at 50 °C above the T_β_ to achieve a 100% β phase microstructure. Moreover, the temperature was kept as low as possible in the β field to prevent excessive grain growth [33]. A similar path was followed by other authors in different works available in the literature [16,25,34]. The samples that underwent a post-annealing aging step (WQ+A and FC+A) were heat-treated at 540 °C for 4 h. This temperature is a standard choice for aging treatments, as reported in other works [35,36].

For each condition, 3 vertically built (Z) cylindrical samples were mechanically machined in order to obtain cylindrical tensile bars, characterized by a gauge length of 40 mm and a diameter of 8 mm, in compliance with ASTM E8 [37]. The specimens were then tested using a ZwickRoell BT1-FR100 testing machine (ZwickRoell GmbH, Ulm, Germany), which operated at a strain rate of 0.008 s^−1^. The yield tensile strength (YTS), ultimate tensile strength (UTS), and elongation (ε) were assessed. During the test, the strain was measured using an extensometer.

A LECO ON736 inert gas fusion infra-red absorption analyzer (LECO, St. Joseph, MI, USA) was used to evaluate the concentration of interstitial elements (oxygen, nitrogen) of the specimens in all the conditions considered in this work. Three samples of approximately 0.1 g each were investigated per each condition.

The cubic samples were cut along the vertical direction and subsequently polished in order to obtain metallographic specimens that were used to investigate the microstructure and to perform X-ray diffraction (XRD) analysis. All the investigations were conducted on cross-sectioned samples oriented parallel to the building direction (Z).

The microstructure of the metallographic specimens was revealed by chemically etching using a Kroll solution (93% H_2_O, 5% HNO_3_, 2% HF). The micrographs were analyzed by using both a Leica DMI 5000M optical microscope (Leica, Wetzlar, Germany) and a Phenom-XL electron microscope (SEM) (Thermo Fisher, Waltham, MA, USA). Different microstructural features were analyzed. To evaluate the α-laths width, 20 micrographs (500× magnification) per sample were considered and analyzed using the ImageJ software. The average β fraction for the samples in the FC and FC+A conditions was measured by image analysis of several SEM micrographs. This operation was performed by appropriately adjusting the threshold of the micrographs using the software ImageJ. This methodology was already successfully adopted by Attallah et al. [38] to investigate titanium alloys. Similarly, in order to study the grains of the material, 100–150 images per sample were taken and stitched together so that the whole surface of the sample was available. Then the average grain size was estimated using the intercept method by creating a grid of lines crossing the whole samples both in the horizontal and vertical direction, approximately 2 mm apart from each other. This methodology was already adopted in other works available in the literature [39,40].

In order to analyze the phase compositions of the specimens, XRD analyses were performed on all the conditions using a PANalytical X-Pert Philips diffractometer (PANalytical, Almelo, The Netherlands). The instrument was set to work with a Cu Kα radiation at 40 kV and 40 mA in a Bragg Brentano configuration. A 2θ range from 30° to 60° and a step size of 0.013° were used. To further analyze the data provided by the XRD evaluation, the determination of the cell parameter for the α phase was conducted. To do so, the hexagonal lattice of this phase was indexed using Bragg’s law. In order to minimize the effect of texturing, the “intensity averaging” method was used, which implies that each parameter was derived from multiple peaks of the pattern [41].

## 3. Results and Discussion

### 3.1. Mechanical Properties and Fracture Surfaces

**Figure 2:**
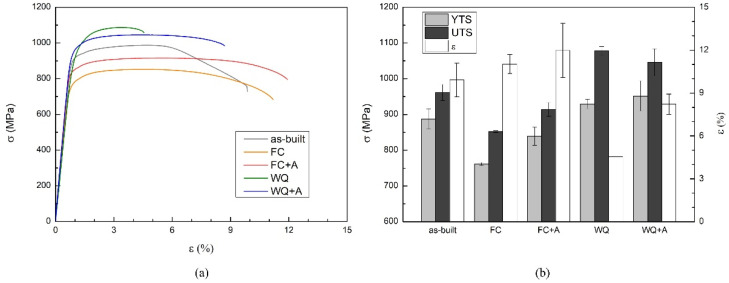
Tensile curves of a representative sample of each condition (**a**) and average YTS, UTS, and ε of all specimens (**b**).

The tensile properties of the as-built and heat-tread samples are reported in Figure 2. The as-built specimens were characterized by average values of YTS, UTS, and ε of 887 ± 28 MPa, 962 ± 23 MPa, and 10% ± 1%, respectively. The first two values were well above the minimum threshold imposed by ASTM-F2924 [42], which describes the standard specification for the Ti-6Al-4V alloy produced via powder bed fusion. The elongation was very close to the minimum requirement (10%). Furthermore, the as-built specimens provided significant necking phenomena after an elongation value of approximately 6% was reached during the test.

**Figure 3:**
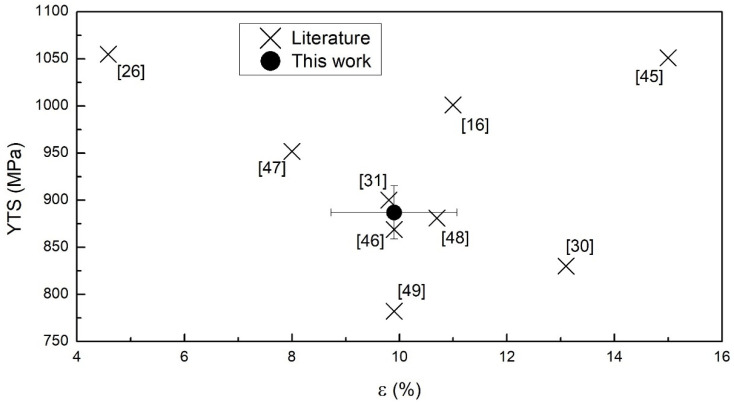
Graphical comparison of the tensile data found in the literature with the tensile properties measured in this work for vertically EBM-produced and mechanically machined samples in the as-built condition [16,26,30,31,45,46,47,48,49].

The tensile properties of the as-built samples were also related to similar samples (vertically built and machined) presented in other works. The aim of this comparative analysis was to consider how the mechanical properties of the specimens in this study fitted with the current literature (Figure 3). To do so, the YTS and ε were investigated. Both of these values appeared as intermediate with respect to the ones provided by other authors. A relevant variability was detected, which might be attributed to the choice of the adopted process parameters and/or possible differences in the quality and properties of powders used. In fact, the adopted energy input greatly influences the tensile properties [43]. The quality of the feedstock material is another important factor that heavily influences the achievable tensile properties [44]. For instance, among the data used for Figure 3, Zhai et al. [45] achieved an optimal combination of mean strength (1051 MPa) and elongation (15%) due to the generation of a very fine lamellar microstructure. Conversely, de Formanoir et al. [26] obtained rather strong specimens (YTS = 1055 MPa) characterized by inferior elongation values (<5%). The authors attributed this effect to oxygen pick-up phenomena during the EBM process. A completely different outcome was achieved by Facchini et al. [30]: their specimens provided low strength (830 MPa) and high deformability (ε = 13.1%). This behavior was probably caused by the rough microstructure induced by the manufacturing process, thus different process parameters.

Considering the heat-treated samples (Figure 2), the FC and FC+A specimens provided the highest ductility values, 11% ± 1% and 12% ± 2%, respectively. Usually, this is linked to a decrease in YTS, as confirmed by the FC samples, characterized by a YTS of 762 ± 4 MPa, which was markedly lower than the as-built condition. However, the FC+A samples provided a YTS of 839 ± 25 MPa, close to the YTS of the as-built samples. The WQ specimens were characterized by the highest UTS measured in this work (1079 ± 12 MPa). The YTS was also superior to the as-built condition (930 ± 13 MPa); instead, the ductility resulted markedly lowered (4.5% ± 0.1%). The WQ+A samples were characterized by a YTS and UTS of 952 ± 42 MPa and 1046 ± 38 MPa. After aging, the ductility reached 8 ± 1%, almost double the ductility of the WQ condition. Overall, the aging treatment proved to be markedly beneficial for both conditions. In the FC+A samples, an almost complete recovery of the strength loss caused by the FC treatment was achieved. Furthermore, a slight increase in terms of plasticity was also obtained. This result appeared particularly promising, as usually, a strengthening effect is associated with a ductility decrease and vice versa. In the WQ+A specimens, a significant recovery, in terms of ε, was possible without significantly reducing the superior strength values obtained via WQ treatment. By comparing the stress–strain curves of the heat-treated and the as-built specimens, the latter type of samples provided a sudden σ decrease after the UTS was reached (necking). This marked behavior was evident only in the as-built condition. The authors attributed this effect to the columnar grain morphology expected, absent in the other specimens due to the recrystallization heat treatments. This morphology resulted in longer and more complex paths for the cracks generated during uniaxial loading since their propagation has a consistent transgranular component [50]. Thus, this effect resulted overall in an improvement of ductility during tensile testing.

The concentration of the interstitial elements (O, N, H) was determined (see Appendix A), to understand any possible influence on the mechanical properties.

**Figure 4:**
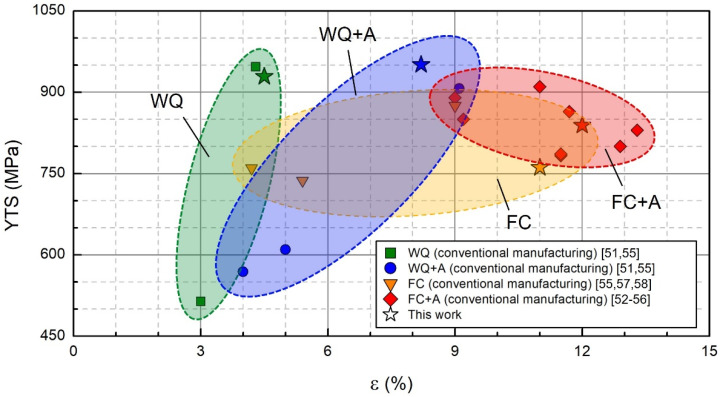
Comparison of the tensile properties of conventionally-manufactured Ti-6Al-4V samples that underwent comparable heat treatments with the specimens part of this study (highlighted with the star symbol) [51,52,53,54,55,56,57,58].

A direct comparison with the literature for heat treatments similar to this work was not possible since no data were found regarding the effect of an aging step on the mechanical properties of super β-transus annealed Ti-6Al-4V EBM-produced samples. Therefore, the mechanical properties of Ti-6Al-4V specimens produced using traditional manufacturing technologies (e.g., casting, forging) that underwent comparable heat treatments were considered and compared with the outcome of this work. The time and temperature ranges of the annealing heat treatments considered were 20 min–2 h and 1000–1100 °C, respectively. For the aging step, these parameters ranged from 5 min to 24 h and from 540 to 950 °C. The outcome of this comparative evaluation is illustrated in Figure 4. According to the collected data from the literature, the post-annealing aging heat treatment seems to provide an overall increase in mechanical properties (YTS, ε) with respect to the un-aged samples.

Similarly, the EBM-produced samples of this work followed the same trend. In fact, the aged Ti-6Al-4V specimens showed an increment of ductility and YTS. For the WQ condition, this was particularly accentuated since the ductility almost doubled its value along with a slight increment of the average YTS. These behaviors reflect the evolution of the tensile properties showed by the EBM-produced samples part of this work, thus confirming the beneficial effect of the aging treatment, even if the manufacturing technology is changed.

**Figure 5:**
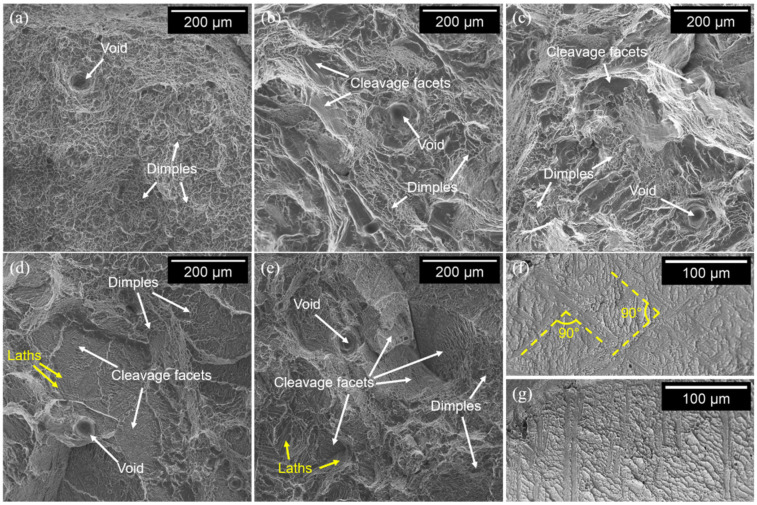
Fracture surface of the as-built (**a**), FC (**b**), FC+A (**c**), WQ (**d**), and WQ+A (**e**) samples; microstructural features visible on the cleavage facets in the WQ (**f**) and WQ+A (**g**) samples.

The fracture surfaces of the as-built samples (Figure 5a) were characterized by the presence of dimples throughout the whole surface and some voids, possibly generated by the coalescence of micropores, indicating extended ductile deformation. This ductile fracture mode in Ti-6Al-4V EBM-produced samples was also reported by other authors [44]. The FC and FC+A samples (Figure 5b,c) provided similar features; however, a relevant number of quasi-cleavage facets was detected, suggesting a brittler behavior. Therefore, the compresence of dimples, microvoids, and cleavage facets suggested a mixed ductile-brittle fracture mode. This result seems counterintuitive when comparing these specimens with the ones in the as-built state since the FC and FC+A samples provided higher ε values during tensile testing (Figure 2). However, these types of fractures are extensively reported in the literature for lamellar microstructures [57,59]. Additionally, coarse α microstructures induce cleavage deformation, whilst fine α laths promote dimples initiation [60]. It is therefore theorized that the fracture mode variation detected was caused by the different microstructures involved. In the end, the WQ and WQ+A samples (Figure 5d,e) were characterized by the presence of smaller dimples. Moreover, the cleavage facets were significantly more numerous and bigger, especially in the WQ specimens, suggesting a more brittle fracture mode with respect to the previous samples. Furthermore, distinct microstructural features were clearly visible on the cleavage facets. These were arranged in a ±45° pattern in the WQ samples (Figure 5f), suggesting being α’ laths, and in a parallel fashion in the WQ+A samples (Figure 5g), suggesting that they are α laths. Moreover, no dimples were found in the correspondence of such features. The presence of martensitic needles on the cleavage surfaces was also noted by Krakhmalev et al. [61] on SLM-produced Ti-6Al-4V samples. Overall, the limited presence of dimples in the WQ and WQ+A specimens confirmed the more brittle behavior provided during the tensile tests. 

### 3.2. Microstructure Investigation

**Figure 6:**
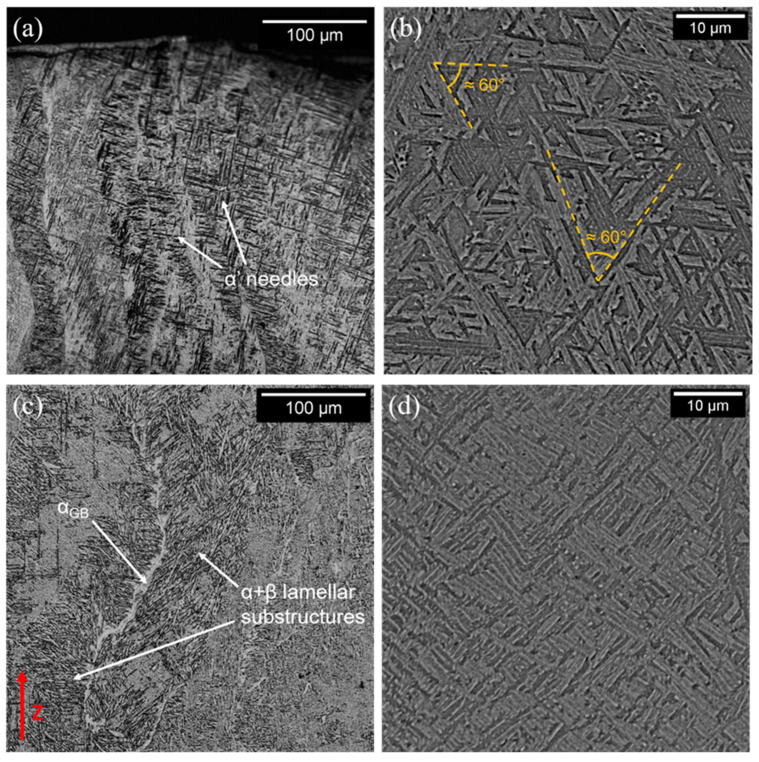
Optical micrographs of the top (**a**) and middle (**c**) parts of the as-built samples. High-magnification SEM images, highlighting different lamellar substructures (**b**,**d**).

The as-built specimens provided in Figure 6 showed a very complex microstructure. This is primarily caused by the complex thermal cycles that the material undergoes during fabrication. In particular, the chamber is maintained at relatively high temperatures, thus acting as a prolonged annealing heat treatment [13,16]. The prior-β grains were developed in a columnar morphology, a typical outcome for EBM-processed Ti-6Al-4V, resulting from the highly directional cooling typical of this process [25,28,62]. A continuous layer of α phase (α_GB_), nucleated at the prior-β grain boundaries, can be observed in Figure 6c. This microstructural feature was also detected by other authors working on EBM-processed Ti-6Al-4V [26,63]. The α+β lamellae were arranged in different substructures within the grains, as highlighted in the examples in Figure 6b,d. For instance, the α lamellae, enveloped in a continuous β layer, were found to be arranged in a Widmanstätten pattern (Figure 6b), recognizable by the typical 60° angles between neighbor lamellae [7]. Lamellar colonies were also found, frequently in correspondence with α_GB_, which aided the formation of such structures. Furthermore, in correspondence of the last layers, traces of completely martensitic α’ grains were found (Figure 6a). A similar microstructural feature was also highlighted by Popov et al. [64]. The presence of such microstructure is correlated to the very high thermal gradients and cooling rates [2,4]. Its localization exclusively in the last layers might have been correlated to the intrinsic annealing of the EBM process, which usually guarantees the α’ → α+β decomposition. However, the upper layers were confined in the process chamber for a shorter time when compared to the ones underneath, thus undergoing a shorter “heat treatment”, which lacked the required duration in order to allow the decomposition of the martensite. Traces of martensite were also found by other authors [65,66].

**Figure 7:**
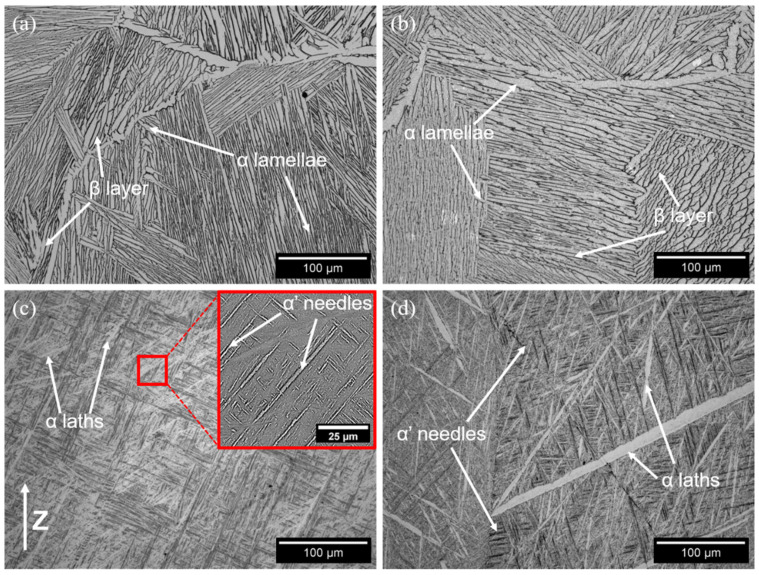
Representative micrographs of the FC (**a**), FC+A (**b**), WQ (**c**) and WQ+A (**d**) samples.

The investigation of the heat-treated samples provided different types of microstructures, characterized by an equiaxed grain morphology, differently from the as-built condition. The microstructure of the FC samples (Figure 7a) was formed by α+β lamellae grouped in colonies, thus sharing the same orientation variant of the relation between α and β phases [67]. This is a typical outcome for a recrystallization heat treatment in which a slow cooling rate is applied, thus providing only a limited number of possible lamellar orientations [67]. It is well known in titanium metallurgy that lamellar α+β microstructures where the α laths are grouped in colonies are usually associated with high ductility values [2,7]. In fact, both the FC and FC+A specimens were characterized by a higher ε during the tensile tests (Figure 2). The FC+A sample (Figure 7b) showed a variation in terms of the β fraction compared to the FC condition. The FC specimens provided an average of 18.1% ± 0.9% β, which decreased significantly to 12.9% ± 1.7% after aging. These data are in good agreement with other works found in the literature [68,69]. The variation in β fraction is related to the β → α+β diffusive transformation that occurs during cooling from above T_β_. Even if the cooling rate achieved at the end of the heat treatment was rather slow, it was insufficient to achieve a completely thermodynamically stable microstructure in terms of phase equilibrium. During aging, the decomposition of the retained β was promoted, granting a higher α fraction. This variation can effectively explain the slight strengthening effect that occurred after aging. In fact, the α phase, more present in the FC+A specimens, is comparatively stronger [70,71]. By contrast, β lowers the YTS and has a beneficial effect on ε [72]. Furthermore, the presence of large α laths in the specimens in the FC and FC+A conditions confirms the assumptions made when comparing the fracture surfaces of these specimens with those in the as-built state (Figure 5). It is then confirmed that the presence of such microstructural features induced the cleavage-like fracture mode.

The WQ samples were characterized by a completely different microstructure, mainly composed of α’ martensitic needles, arranged in the typical ±45° fashion (Figure 7c) [2], as also seen on the cleavage facets of the fracture surfaces (Figure 5f). Moreover, small traces of more thermodynamically stable α laths were found. The presence of α’ can effectively explain the strengthening effect detected during the tensile test (Figure 2) [2,73]. The formation of this particular type of microstructure is caused by applying a very high cooling rate (>410 °C/s) from T > martensite start (MS), which is approximately 800 °C for Ti-6Al-4V [23,74]. Martensite formation is usually avoided, as α’ laths are very effective at hindering dislocation motion, thus embrittling the material. In fact, the slip length of α’ is usually limited to a single grain, thus favoring dislocations pile-up. Instead, in duplex lamellar microstructures, slip transfer is allowed between different phases, increasing mobility dislocation [75]. 

Instead, the WQ+A samples (Figure 7d) still showed a mainly martensitic microstructure, but the α laths resulted more numerous and visibly larger in size, possibly at the expense of α’. Since diffusion phenomena are involved in this type of transformation (α’ → α+β), this microstructural variation is greatly favored by the long-aging heat treatment. The partial decomposition of the martensite is in good agreement with the improved plasticity detected during the tensile test. Moreover, the limited disappearance of α’ in favor of α was also confirmed by the presence of the latter on the cleavage facets observed on the fracture surfaces (Figure 5g). The enhanced plasticity in the WQ+A specimens can easily be related to the intrinsic properties of the α and α’ phases. It was expected that such a relevant ε increase with respect to the unaged condition (+89%) would be coupled with a severe loss in YTS. However, this phenomenon did not occur, possibly due to a shift in the relative fractions of α and α’. These fractions are directly correlated to the number of α/α’ interfaces in the material, which play a key role in the determination of the tensile properties. In fact, during uniaxial loading, the stress accumulates in correspondence with these interfaces, which are very prone to accommodating dislocation generation. This results in an overall strengthening effect, which is maximized if the α/α’ ratio is close to 0.5, enhancing the number of relative interfaces [76]. In this work, the WQ samples were characterized by a very low α/α’ ratio due to the almost complete lack of α laths. Instead, the aging process resulted in α generation and martensite reduction, shifting the ratio towards higher values. The resulting strengthening effect may have compensated for the strength loss caused by the partial disappearance of the strong martensite, resulting in a slight overall YTS reduction. In this study, the assumptions related to the shift of the α/α’ ratio were made without considering the size effect. In fact, the number of interfaces available is strictly dependent on the size of the α and α’ laths. In this specific case, the authors did not take into account this phenomenon, as it appeared negligible, due to the clear overabundance of α in the WQ+A samples, with respect to the WQ specimens.

**Figure 8:**
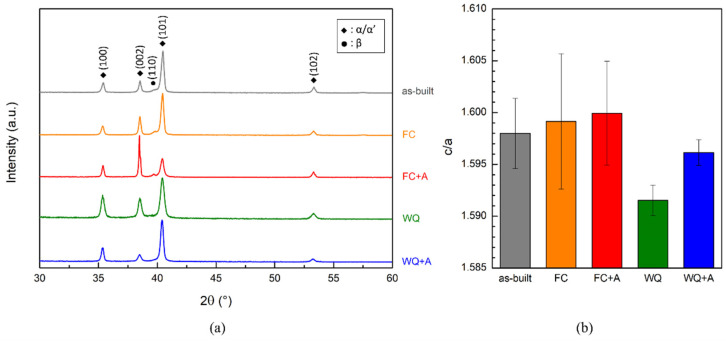
XRD patterns for all the considered conditions evaluated along the building direction (**a**) and relative c/a ratio values (**b**).

XRD analyses were conducted on all the samples (Figure 8a). All the specimens showed the peaks relative to α/α’. These two phases are hardly completely distinguishable, as they share a common hexagonal structure [77,78]. However, only the as-built and furnace-cooled samples provided the (110) peak relative to the β phase. In general, α formation upon cooling also implies the formation of the β, whether it nucleates from high-temperature β or from α’ decomposition. Therefore, when the β peak is present, the microstructure is formed by stable α instead of metastable martensite. In contrast, since the martensitic transformation β → α’ does not allow the generation of the β phase, the microstructure is likely to be mainly martensitic if this peak is absent. Both of these assumptions are in good agreement with the results obtained from the investigation of the microstructure. In fact, the as-built FC and FC+A samples provided a duplex α+β microstructure, whilst the WQ and WQ+A specimens showed the presence of α’, recognized by the typical ±45° oriented needles [2]. The FC+A specimens were characterized by a (002) α/α’ peak higher than the (101) peak, unlike all the other samples. The authors of this work attributed this phenomenon to texturing. 

To further investigate the microstructure, the determination of the cell parameters related to the α/α’ phases were calculated from the c/a ratio provided by the XRD data (Figure 8b). The as-built FC and FC+A samples provided similar c/a average values, ranging from 1.598 to 1.600. These values were consistent with others found in the literature for α+β microstructures [79,80]. On the contrary, the WQ and WQ+A specimens provided smaller c/a ratios, in particular WQ (1.5915 ± 0.0015). This distortion of the cell might be correlated to the presence of martensite, which is supersaturated with β stabilizing elements, unlike α [81,82]. A lower c/a is typical for a martensitic Ti-6Al-4V microstructure [39,83]. For instance, Malinov et al. [83] evaluated the lattice parameters of Ti-6Al-4V samples that underwent different sub-β and super β-transus heat treatments. In the last case, the specimens that were water quenched at the end of these operations provided only the peaks relative to the α/α’ phases in their XRD pattern, suggesting a mainly martensitic microstructure. Moreover, Cho [84] also suggested that a lower c/a ratio in titanium alloys is a good indicator of the presence of martensite. 

The c/a ratio of FC and FC+A conditions were similar to each other, indicating that the α and β phases were thermodynamically stable. In contrast, a significant increase in the c/a value occurred after aging in the WQ samples due to the partial decomposition of the martensite occurring, thus generating α and relaxing the cell distortion.

**Figure 9:**
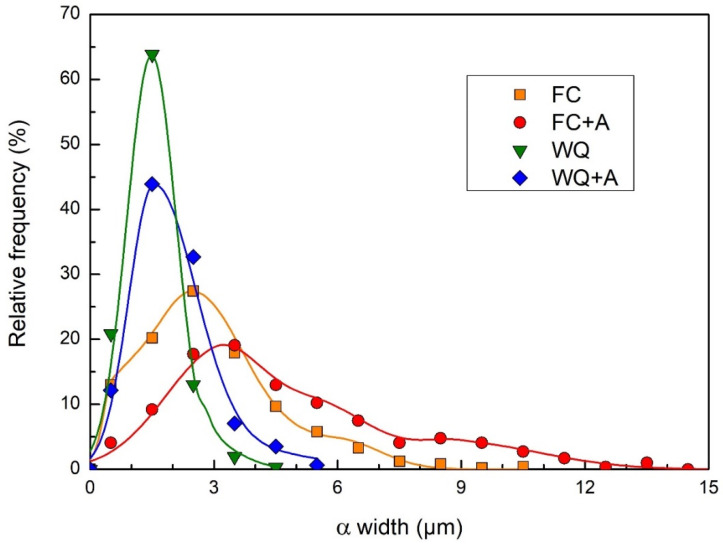
Relative distribution of the α width for all the heat-treated samples. The same step size was used in all the curves for comparability reasons.

In order to conduct a more in-depth microstructural characterization, the evaluation of the width of the α laths for all the heat-treated samples was conducted. This microstructural feature was chosen as commonly shared by the specimens in all the analyzed conditions. The as-built samples were not considered in this analysis since their microstructure, in which several types of morphologies (colonies and basket-weave) and phases (α, β, α’) exist simultaneously, was inhomogeneous, thus rather incorrect to describe using a single microstructural parameter. In fact, average values ranging from 0.2–0.5 µm (basket-weave morphology) to 3–6 µm (lamellar colonies) were measured. These resulted in rather inhomogeneous distributions, highly dependent on the areas considered. Average α width values well below 1 µm were measured by several authors [9,85]. However, α width can also reach an order of magnitude larger, as reported in the literature [86]. For these reasons, the results of the evaluation provided in Figure 9 do not report the curve relative to the as-built condition.

The FC and FC+A samples were characterized by much larger α laths. In fact, they provided an average value of 2.86 and 4.72 µm, respectively. The FC condition resulted in comparable data with similar heat treatments (1000–1100 °C, 1–2 h, FC) performed on EBM-produced samples available in the literature. In these studies, lamellar microstructures, characterized by average α laths ranging from 2 to 2.5 µm in width approximately, were detected [25,87]. As already mentioned, no references for the FC+A heat treatment performed on EBM-produced samples were found in the literature. Overall, the aging step provided significant coarsening of the lamellar microstructure. This microstructural effect can be associated with the slightly improved plasticity of the FC+A samples with respect to the FC specimens. In fact, a rougher lamellar microstructure (α laths thickness) leads to improved plasticity values [7], thus reducing the YTS. However, the strength is also correlated to the grain size and α and β relative fractions [69]. It was therefore theorized that two opposite outcomes took place simultaneously in the FC samples after aging: the β fraction reduction produced a strengthening effect, which increased YTS and reduced ε. Parallelly, the lamellar microstructure was roughened, i.e., increasing α laths thickness, which increased the plasticity and possibly decreased the strength of the material. The overlap of these two phenomena leads to an overall increase in YTS and ε simultaneously. Instead, the WQ and WQ+A samples provided average α widths of 1.46 and 1.98 µm, respectively. Of course, the α laths analyzed were part of two different morphologies (lamellar α+β and acicular α’+α laths), resulting from markedly different thermal histories. Hence, their comparison only demonstrates that the growth of the α phase, which is diffusion-driven, is greatly favored when low cooling rates are applied. In fact, the prolonged duration of the heat treatment, caused by the long time needed for the system to cool down, better accommodates diffusive phenomena. When comparing the aged and unaged conditions of both groups (FC and WQ), α growth was evident during the aging heat treatment, which then had a great impact on the final microstructure of the specimens analyzed. This is particularly evident when comparing the optical micrographs of the water-quenched samples (Figure 7c,d). In fact, the representative WQ+A microstructure appeared evidently rougher and richer in terms of the α phase.

**Figure 10:**
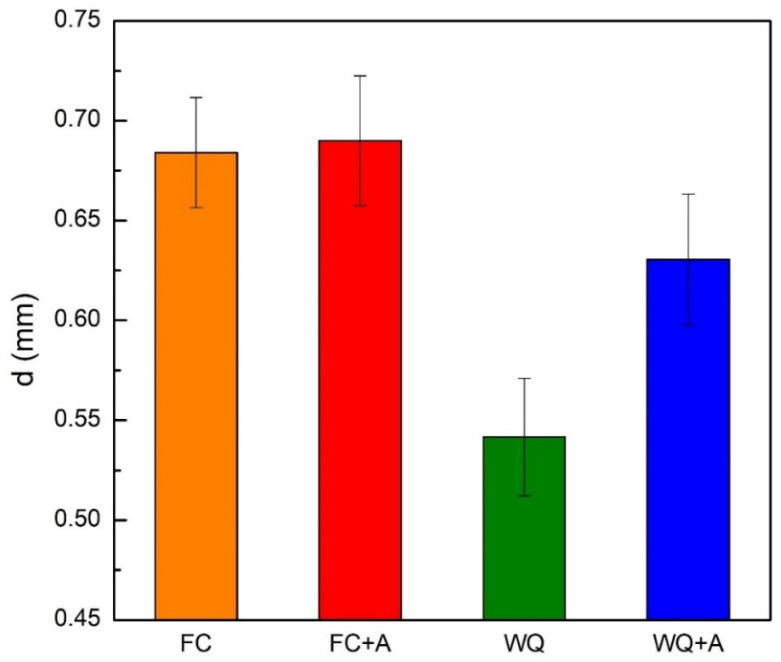
Grain size evaluation for all the heat-treated samples.

Prior-β grain size (d) is another critical parameter to evaluate since it has a direct impact on the mechanical properties of the final component [88,89]. It was then evaluated only in the heat-treated samples since observation of grains via optical means is a challenging task, as also reported by Galarraga et al. [90]. Moreover, a direct comparison with the as-built specimens is difficult due to the different grain morphologies (columnar and equiaxed). The results of the evaluation (Figure 10) showed a marked variation of this parameter according to the heat treatment that the specimens underwent. The FC and FC+A samples provided the largest grains, consistently with the microstructure evaluation, since grain enlargement is a diffusion-driven phenomenon, similar to α growth [2,91]. Moreover, all the annealing treatments were conducted above T_β_, thus at a temperature in which the sample is 100% β phase, and the self-diffusivity coefficient for β titanium (D_Ti-β_ ≈ 10–13 m^2^/s) is much greater than that of α titanium (D_Ti-α_ ≈ 10–15 m^2^/s) [33]. Considering that, the super β-transus heat treatments must be carefully controlled in terms of duration and temperature, as the lack of α phase, much more effective at preventing excessive grain growth, can lead to the formation of grains excessively coarse. The difference in terms of d between FC and FC+A samples was negligible. This was correlated to the presence of α_GB_, formed during the cooling phase of the annealing (Figure 7c). This microstructural feature is very effective at hindering grain enlargement [26]. Instead, the WQ samples provided much finer grains due to the extremely fast cooling rate applied, which prevented diffusive phenomena from happening during cooling. However, the annealing led the WQ+A samples to provide larger grains, as no barrier (α_GB_) was present, unlike in the previous case. Thus, in this case, the aging step provided microstructural coarsening, both in terms of lamellar and grain sizes. The grains evaluated in this work ranged from approximately 0.5 to 0.7 mm in size, according to the cooling path followed. The analysis of the literature for similar heat-treated conditions of EBM-produced samples (1000–1050 °C for 1–2 h) provided significantly different values, ranging from 0.3–0.4 mm up to more than 1 mm [25,26,29]. Overall, the evaluation of d was in good agreement with the tensile properties obtained for the heat-treated samples (Figure 2). In fact, improved ε values were correlated with larger grains. Thus, the FC and FC+A samples showed the highest elongation among the different conditions. Considering the WQ and WQ+A specimens, an increase in ductility was also recorded after the aging step. This was also partially associated with grain roughening, in conjunction with the decomposition of the embrittling martensitic phase. The related YTS loss was probably compensated by the variation in the number of α/α’ interfaces. 

To sum up the correlation of the tensile properties with the microstructural features, the samples in the FC condition provided a completely lamellar microstructure with visible colonies. This microstructure led to a decrease in terms of YTS and UTS but an improvement in ε with respect to the as-printed state. For these specimens, the average grain size was unaffected by aging, as α_GB_ acted as a diffusion barrier, suppressing inter-grains diffusive paths. Instead, the aging step (FC+A condition) resulted simultaneously in significant coarsening of the α phase (lamellar enlargement) and a decrease in the β fraction. The coarsening of the α phase is expected to result in improved plasticity and reduced strength. Instead, the decrease in the β fraction provided an opposite outcome. All of these effects resulted in a further improvement of plasticity, linked with a slight strengthening effect with respect to the FC condition.

For the WQ condition, a martensitic (α’) microstructure with traces of α was achieved. This led to a significant increase in YTS and UTS, at the cost of a severe reduction in ε, with respect to the as-built state. In this condition, the aging step (WQ+A condition) simultaneously provided a partial decomposition of α’ and the subsequent nucleation of α. Moreover, the pre-existing α lamellae experienced growth phenomena. In terms of grain size, the combination of high temperature and long duration of the aging step promoted diffusion, causing grain coarsening, which is usually related to an improvement in plasticity at the cost of YTS reduction. However, the increase in α content caused a significant increment in the α/α’ ratio and the relative number of interfaces between these two phases, resulting in an overall strengthening effect, which might have prevented a conspicuous drop in YTS. All of these phenomena resulted in a small decrease in strength, associated with an almost complete recovery of the strength loss caused by water quenching.

## 4. Conclusions

This work assessed the influence of different super β-transus annealing heat treatments characterized by different cooling rates, with and without a post-annealing aging step, on the microstructural and mechanical properties of the Ti-6Al-4V alloy produced using EBM. Overall, the super β-transus annealing heat treatments provided a shift from a columnar to a completely equiaxed grain morphology, with improved isotropy. Furthermore, aging the samples proved to be beneficial for all the conditions considered, specifically in terms of plasticity enhancement. The most important results for each condition are reported as follows:

- the furnace-cooled samples showed a slightly improved ductility and a drop in strength due to the lamellar colonies generated during the heat treatment.
- after aging, the furnace-cooled samples provided small increases in terms of strength and ductility due to the simultaneous α coarsening and β fraction reduction.
- the water-quenched samples resulted significantly strengthened and embrittled due to the presence of martensite as a result of the adopted cooling path.
- after aging, the water-quenched samples experienced a slight strength drop and a significant ductility improvement due to simultaneous grain coarsening and α growth/nucleation phenomena.

## Figures and Tables

**Table 1:** Conditions considered and relative heat treatments specifications.

Condition	Name	Annealing	Aging
As-built	as-built	-	-
Furnace Cooled	FC	1050 °C for 1 h, then FC	-
Furnace Cooled + Aged	FC+A	1050 °C for 1 h, then FC	540 °C for 4 h, then FC
Water Quenched	WQ	1050 °C for 1 h, then WQ	-
Water Quenched + Aged	WQ+A	1050 °C for 1 h, then WQ	540 °C for 4 h, then FC

## Data Availability

Not applicable.

## Appendix A

Oxygen and nitrogen are interstitial elements in α titanium. Their content must be kept as low as possible, as these elements can significantly embrittle the alloy. ASTM F136 allows a maximum concentration allowed for these elements of 0.13% and 0.05% for oxygen and nitrogen, respectively. These values were measured for all the conditions considered in this work. The results of the analysis, reported in Table A1, confirm that no significant oxygen/nitrogen pick-up occurred during the heat treatments. Therefore, the comparative analysis of the tensile properties can be conducted without taking into consideration this effect.

**Table A1:** Oxygen and nitrogen contents.

Condition	Oxygen Content (%)	Nitrogen Content (%)
As-built	0.097 ± 0.001	0.012 ± 0.002
FC	0.092 ± 0.012	0.014 ± 0.007
FC+A	0.103 ± 0.001	0.018 ± 0.003
WQ	0.099 ± 0.003	0.016 ± 0.002
WQ+A	0.096 ± 0.004	0.016 ± 0.005
